# The Role of T Cell Senescence in Neurological Diseases and Its Regulation by Cellular Metabolism

**DOI:** 10.3389/fimmu.2021.706434

**Published:** 2021-07-14

**Authors:** Johannes Fessler, Stefano Angiari

**Affiliations:** Division of Immunology and Pathophysiology, Otto Loewi Research Center, Medical University of Graz, Graz, Austria

**Keywords:** immunosenescence****, T cell, neuroinflammation, neurodegeneration, immunometabolism

## Abstract

Immunosenescence is a state of dysregulated leukocyte function characterised by arrested cell cycle, telomere shortening, expression of markers of cellular stress, and secretion of pro-inflammatory mediators. Immunosenescence principally develops during aging, but it may also be induced in other pathological settings, such as chronic viral infections and autoimmune diseases. Appearance of senescent immune cells has been shown to potentially cause chronic inflammation and tissue damage, suggesting an important role for this process in organismal homeostasis. In particular, the presence of senescent T lymphocytes has been reported in neurological diseases, with some works pointing towards a direct connection between T cell senescence, inflammation and neuronal damage. In this minireview, we provide an overview on the role of T cell senescence in neurological disorders, in particular in multiple sclerosis and Alzheimer disease. We also discuss recent literature investigating how metabolic remodelling controls the development of a senescence phenotype in T cells. Targeting metabolic pathways involved in the induction of senescent T cells may indeed represent a novel approach to limit their inflammatory activity and prevent neuroinflammation and neurodegeneration.

## Introduction

Aging is a natural process that has to deal with a multitude of challenges, and loss of organismal homeostasis during aging is frequently associated with increased susceptibility to infection, cancer, cardiovascular disease and autoimmunity in the elderly (1–4). A dysregulation of the immune system, ‘termed immunosenescence’, is a hallmark of the aging process, and is thought to play a central role in the higher likelihood of developing such pathologies. Senescent immune cells display a dysfunctional immune profile, including altered cytokine production, limited proliferative capacity and reduced chemotactic and phagocytic potential (5). Immunosenescence is typically associated with chronological age, and it is tightly connected to the process of ‘inflammaging’, i.e. the chronic and systemic low-grade inflammation observed in the elderly (6). However, premature immunosenescence, in particular in T lymphocytes, has also been observed in patients with chronic viral infections and in autoimmune diseases (7–10). Thus, it is essential to appreciate those influences in the context of immunosenescent manifestations.

Age represents the main risk factor for the development of several neurological disorders, in particular chronic neurodegenerative pathologies such as Alzheimer disease (AD) and Parkinson disease (PD), where inflammaging is proposed to play a relevant role in the disease course (11). Blood-borne immune cells infiltrating the central nervous system (CNS) and causing direct or indirect neuronal damage also have a central role in CNS pathologies such as multiple sclerosis (MS), traumatic brain injury (TBI), ischemic stroke, AD and PD (12–16). Among immune cells, T lymphocytes represent key players in neurological diseases, where they cause detrimental inflammation taking place in the CNS, but also participate in regulatory mechanisms aimed at protecting neurons from the inflammatory damage (12–16). Intriguingly, an increasing number of studies reported the presence of T cell subsets with a senescent-like phenotype in patients with neurological disorders (senescent T cells, sTC). Here, we summarise our knowledge on the role of sTC, in particular CD4^+^ and CD8^+^ conventional T cells, in neuroinflammation and neurodegeneration. We also provide an overview of recent works showing how intracellular metabolic reprogramming may modulate the development of a senescent phenotype in T lymphocytes.

## T Cell Senescence in Neurological Diseases

**Figure 1:**
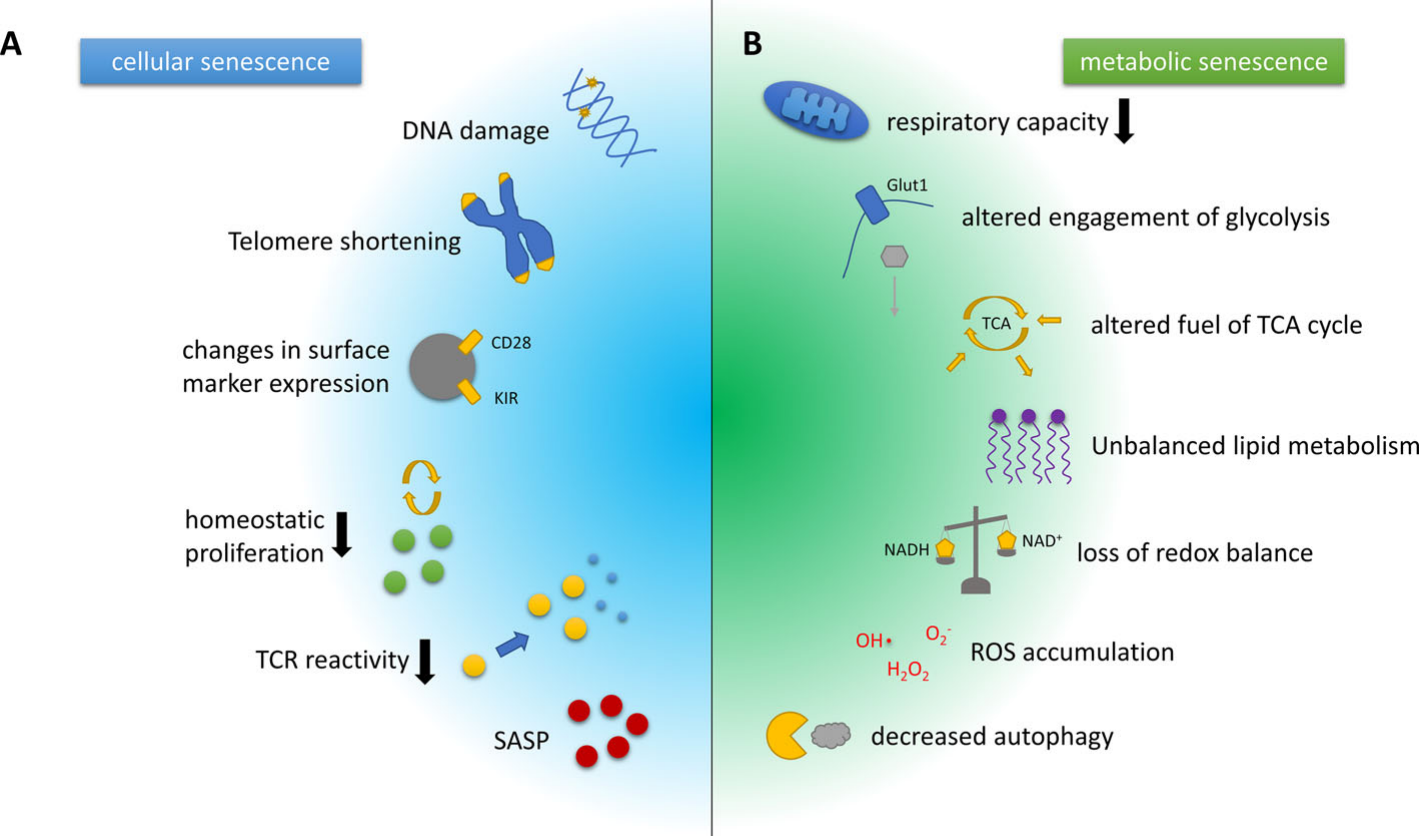
Hallmarks of cellular and metabolic senescence and/or aging in T cells. **(A)** Key characteristics of cellular senescence include DNA damage and telomere erosion that contribute to genomic instability and dysregulation of the epigenome. Phenotypically, T cell senescence is associated with a loss of surface CD28 expression and the upregulation of innate T cell markers such as the killer cell immunoglobulin-like receptors (KIRs). The involution of the thymus increases the homeostatic pressure on T cells. Senescent T cells, however, show a diminished capacity for homeostatic proliferation, and additionally feature reduced T cell receptor (TCR) reactivity. Another hallmark of T cell senescence is the production of pro-inflammatory mediators, collectively known as senescence-associated secretory phenotype (SASP). **(B)** Key metabolic characteristics associated with T cell senescence and aging often closely interwoven and include a decline in the respiratory capacity or efficacy and substantially altered engagement of glycolysis, as well as alterations in the pathways used to fuel the tricarboxylic acid (TCA) cycle, which are likely context- and cell subset-dependent. The same holds true for the dysbalanced lipid metabolism observed in senescent/aged T cells. In addition, ratios of coenzymes for metabolic reactions such as NAD/NADH that are crucial for balancing the cellular redox state are shifted in senescent cells. Furthermore, reactive oxygen species (ROS) accumulate and eventually cause DNA and protein damage. Damaged organelles, cell membranes and proteins are usually degraded by autophagy, another pivotal mechanism that is attenuated by the aging or the senescent process.

T cell senescence is characterised by a decline in naïve T cell number and clonal diversity, which are mainly caused by age-associated thymic atrophy and reduced homeostatic proliferation of naïve-resting T cells. sTC also show loss of their proliferative capacity upon T cell receptor (TCR) reactivation, accelerated telomeric erosion, and accumulation of DNA damage. Throughout the development of a senescent phenotype, T cells downregulate co-stimulatory markers like CD28, while up-regulating natural killer cell-associated molecules, including the killer lectin receptor G1 (KLRG1) (8, 17, 18). These alterations induce a refractoriness of sTC to TCR-mediated activation, but, in parallel, they potentially augment antigen-independent effector functions. Finally, sTC display a pathogenic phenotype characterised by the secretion of several pro-inflammatory mediators, such as the cytokines tumour necrosis factor-alpha (TNF-α) and interferon-gamma (IFN-γ), collectively known as senescence-associated secretory phenotype (SASP) (8, 17, 18) (**Figure 1A**). sTC may thus support the systemic low-grade inflammation observed in the elderly, i.e. inflammaging. However, to what extent sTC are involved in neurological diseases is still uncertain.

Development of a senescent phenotype in T cells may be relevant in MS, an autoimmune disease characterised by CNS infiltration of peripherally activated immune cells that cause neuroinflammation, neuronal death and disability (19). In particular, myelin-specific T cells are key players in the disease, and recent works highlighted the potential involvement of sTC, in particular CD4^+^ T cells, in MS (**Table 1**). Accelerated thymic involution was reported in MS patients with either relapsing-remitting (RRMS) or primary progressive (PPMS) MS forms (20), and initial works also found an increased percentage of CD4^+^CD28^-^ T cells in a subset of MS patients (mainly RRMS), preferentially producing IFN-γ (21). CD4^+^CD28^-^ T cells were subsequently detected in MS brain biopsies, where they displayed a cytotoxic phenotype and expressed CX3C chemokine receptor 1 (CX_3_CR1), which binds to chemokine (C-X3-C motif) ligand 1 (CX3CL1) (22). As CX3CL1 is upregulated in the cerebrospinal fluid (CSF) and brain of MS patients, compared to healthy controls, the authors speculated a CX3CL1-mediated recruitment of these highly inflammatory cells in the MS brain. Importantly, some CD4^+^CX3CL1^+^ brain cells where in close proximity to cleaved caspase-3 (cCASP3)^+^ oligodendrocytes, suggesting that they may cause direct oligodendrocyte death and demyelination (22). Another recent work performed a longitudinal analysis of several markers in T cells from blood, CSF and brain samples obtained post-mortem from patients with advanced disease (mainly progressive MS) (23). Authors found that CD8^+^ T cells in white matter lesions displayed a more chronically activated, effector memory-like profile, compared to their blood counterparts. They also proposed that chronic reactivation in the brain, confirmed by limited TCR diversity in the CD8^+^ cell population, was caused, at least in part, by specific reactivity against Epstein Barr virus-infected B cells (23). These T cells also presented a cytotoxic phenotype, and in some cases they co-localised with cCASP3^+^ cells in the brain, indicating putative cytotoxic activity *in situ*. However, the expression of the senescent marker CD57 was comparable between blood-derived T cells and brain T cells (23), which might exclude induction of cell senescence in such chronically activated, brain-infiltrating T cells.

**Table 1:** Summary of previous works suggesting the presence of senescent T cells in MS and AD patients.

DISEASE	EVIDENCES OF T CELL SENESCENCE	REFERENCES
**Multiple sclerosis**	- Accelerated thymic involution in RRMS and PPMS patients	Reviewed in Haegert DG, Mult Scler Int 2011 (20)
	- Increased percentage of circulating IFN-γ-producing CD4^+^CD28^-^ T cells in a subset of RRMS patients	Reviewed in Broux et al., Trends Mol Med. 2012 (21)
	- CX3CL1-mediated infiltration of potentially cytotoxic CD4^+^CD28^-^ T cells in MS brain	Broux et al., J Autoimmun. 2012 (22)
	- Presence of chronically activated, effector memory-like CD8^+^ T cells with putative cytotoxic activity in white matter lesions of progressive MS patients	van Nierop et al., Acta Neuropathol 2017 (23)
	- Reduced thymic output of naïve T cells and increased percentage of circulating memory-like T cells in paediatric MS patients	Balint et al., Neurology 2013 (24)
**Alzheimer disease**	- Association of telomere shortening in T cells with mild cognitive impairment and dementia in Down syndrome patients	Jenkins et al., Neurobiol Aging. 2006 (25); Jenkins et al., Neurobiol Aging. 2010 (26)
	- Positive correlation of shorter telomere length in T cells with disease severity, plasma TNF-α levels, lower CD28 expression by CD8^+^ T cells, and increased sensitivity to apoptosis in T cells in AD patients	Panossian et al., Neurobiol Aging. 2003 (27)
	- Decreased percentage of naïve CD4^+^ T cells and increased percentage of terminally differentiated memory CD4^+^ T cells expressing KLRG1 in AD patients	Pellicanò et al., J Neuroimmunol. 2012 (28)

T cell senescence was also suggested to play a role in AD, a multifactorial neurodegenerative disorder characterised by progressive neuronal death and development of dementia (**Table 1**). AD has long been viewed as a ‘pure’ neurodegenerative disease, but recent work highlighted the importance of both local and peripheral immune system, including T cells, in disease onset and progression (29, 30). Telomere shortening in T cells, a marker of proliferative senescence, was initially associated with signs of mild cognitive impairment and dementia in Down syndrome patients, which develop premature AD-like dementia (25, 26). Shorter telomere length in T cells was also reported in AD patients, where it directly correlated with disease severity, higher plasma TNF-α levels, lower CD28 expression by CD8^+^ T cells, and increased sensitivity to apoptosis in T cells (27). These data suggest that dysfunctional, senescent T cells may associate with higher disease burden and systemic inflammation in AD. Supporting this view, another work showed decreased percentage of naïve CD4^+^ T cells and increased percentage of terminally differentiated memory CD4^+^ T cells expressing KLRG1 in AD patients, compared to aged-matched controls, while CD8^+^ T cell phenotype was unchanged (28). Lack of CD8^+^ sTC was lately confirmed by another study (31), suggesting that the senescent phenotype might be restricted to CD4^+^ T cells in AD individuals. However, T cell senescence was recently not confirmed in patients with AD or vascular dementia (32), and conflicting results have also been reported in other forms of dementia and neurodegeneration. Indeed, while a trend towards an increased number of circulating senescent-like CD8^+^CD45RA^+^CCR7^-^ T cells was observed in dementia with Lewy bodies (33), a recent work found reduced numbers of senescent/terminally differentiated CD8^+^ T cells in the blood of PD patients (34). Thus, the role of sTC in neurodegeneration and dementia is still unclear.

The involvement of senescent T cells in other neurological conditions is still unknown. Several studies associated signs of leukocyte senescence like telomere shortening, oxidative stress, and reduced lymphoproliferative potential with onset or severity of other neurological disorders, such as PD (35), ischemic stroke (36) and amyotrophic lateral sclerosis (37). However, such works didn’t discriminate between different immune cell populations, and whether T cell senescence plays a role in these diseases has not been investigated. Interestingly, reversed CD4:CD8 ratio, increased percentage of effector memory and reduced numbers of naïve CD4^+^ T cells, all potential signs of T cell senescence, were correlated with the presence of chronic viral infections and cognitive dysfunctions in old individuals (38–40). Similarly, expansion of CD8^+^CD28^-^ T cells was associated with worse cognitive performances in patients with rheumatoid arthritis (41), and higher numbers of memory CD4^+^ T cells and CD8^+^ sTC negatively correlated with cognitive impairment in systemic lupus erythematous patients (42). These works support the idea that persistent T cell activation and maturation observed during chronic viral infections and autoimmune diseases may generate detrimental sTC that cause or sustain cognitive impairment, most likely through pro-inflammatory mechanisms. Additionally, a shift towards a memory/effector-like phenotype in T cells was detected in patients after spinal cord injury (43), possibly caused by the sustained inflammation accompanying the acute damage. Similarly, in a mouse model of TBI, the concussion injury induced an acute lymphopenic response, with reduced thymic size and reduced number of circulating T cells (44). Noteworthy, some of these effects were maintained chronically (60 days after trauma), with T cells also developing into a more pro-inflammatory phenotype and CD4^+^ T cells displaying an effector/memory-like polarisation (44). These studies suggest that acute CNS injury may cause premature T cell senescence, which would eventually sustain the detrimental systemic inflammatory response.

## Metabolic Regulation of T Cell Aging and Senescence

It is now well established that intracellular metabolic remodelling plays a key role in the activation and engagement of effector functions in T cells (45). T cell metabolism has been extensively investigated in the last few years, including the metabolic profile of T cells from elderly individuals, but relatively little is known about the metabolic remodelling controlling T cell senescence (**Figure 1B**). Also, it remains unclear if such changes are a cause of T cell dysfunction or are rather a by-product of the aging process.

Several studies found mitochondrial alterations in senescent lymphocyte subsets or aged T cells (46–50). Ron-Harel and colleagues reported declined mitochondrial mass and reduced basal and maximal respiratory capacity in T cells from old mice (47). Interestingly, aged naïve T cells also showed lower glycolytic activity, as well as low levels of central carbon intermediates in glycolysis, pentose phosphate pathway, and tricarboxylic acid (TCA) cycle, suggesting and overall slower metabolism, respiration and protein synthesis (47). Upon activation, a specific deficit in the induction of enzymes of one-carbon metabolism in aged cells was shown, that potentially accounts for impaired T cell activation, such as the diminished response to vaccination observed in the elderly (47, 51). Another study showed that mitochondrial proteins involved in the electron transport chain were elevated, but at the same time mitochondrial respiration was impaired in total CD4^+^ T cells from older individuals. The authors also noted a significantly higher number of autophagosomes containing undegraded mitochondria, and thus suggested a defective mitochondrial turnover by autophagy, which may trigger chronic inflammation (49). Defective autophagy and mitochondrial bioenergetics in CD4^+^ T cells from older individuals were recently confirmed, and associated with redox imbalance (50). These metabolic alterations furthermore led to a specific proinflammatory profile of IL-17 producing (Th17) cells in older individuals, suggesting that Th17 cells are a pivotal driver of inflammaging. Noteworthy, treatment with the drug metformin enhanced autophagy and normalised mitochondrial function to attenuate age‐associated inflammation (50).

Aged, resting cells feature other major metabolic alterations, such as enhanced basal activation of the phosphoinositide 3-kinase/protein kinase B/mammalian target of rapamycin (PI3K/Akt/mTOR) pathway. Lymphocytes of patients carrying dominant-activating mutations in PI3K exhibited an aged phenotype (accumulation of senescent or terminally differentiated cells), in combination with augmented mTOR signalling and glycolysis, and cellular malfunction (52). Intriguingly, treatment with the mTOR inhibitor rapamycin reversed the immunosenescent and dysfunctional effects in these patients (52). Chronic PI3K/Akt/mTOR pathway activation may also be caused by chronic infections in humans, where T cells upregulate glucose transporter 1 (Glut1) and increase their glycolytic activity, which ultimately leads to CD4^+^ T cell depletion *in vivo* (53, 54). Given that chronic viral infections are known to induce sTC (7), this signalling axis may be relevant in infection-induced T cell senescence. A recent work also reported that T cells from old mice relied heavily on glutaminolysis, and are potentially involved in Th1 and Th17-driven alloimmune responses (55). Treatment with an inhibitor of glutaminolysis prolonged allograft survival specifically in old recipients, whereas in young animals, additional inhibition of glycolysis and oxidative phosphorylation (OXPHOS) was needed to achieve the same effect. Of note, immunosuppressive capacities of glutaminolysis inhibition were specific to CD4^+^ T cells, and depletion of CD8^+^ T cells did not alter transplant outcome (55). Memory CD4^+^ T cells from aged individuals were also shown to upregulate fatty acid β-oxidation (FAO)-coupled mitochondrial respiration, with this process being mediated *via* upregulation of sirtuin-1, a nicotinamide adenine dinucleotide (NAD)^+^-dependent protein deacetylase, that leads to increased carnitine palmitoyl transferase (CPT1a) transcription and maintains a more lipid-catabolic state (56). However, another study showed an age-related loss of sirtuin-1 in human CD8^+^CD28^-^ T cells, that potentially contributes to metabolic reprogramming towards an enhanced glycolytic capacity (57). Interestingly, constitutive activation of glycolytic flux was reported to limit memory development in CD8^+^ T cells. Accordingly, blocking glycolytic metabolism promoted the generation of long-lived, functional memory cells. Enforcing glycolysis, on the other hand, drives CD8^+^ T-cells towards a terminally differentiated state (58). These findings suggest that high glycolytic rates may be involved in the induction of terminally differentiated/sTC.

In an elegant study, Lanna et al. observed that senescent CD4^+^CD27^−^CD28^−^ T lymphocytes exhibit endogenously elevated p38 phosphorylation, triggered by the intracellular metabolic sensor 5’ adenosine monophosphate-activated protein kinase (AMPK) (59). In this context, AMPK responds to endogenous DNA damage and also to a fall of intracellular energy levels, thus highlighting an ‘intra-sensory’ pathway for p38 activation that senses intracellular changes such as glucose deprivation and genotoxic stress. Triggering this pathway leads to inhibition of T cell proliferation and telomerase activity, two typical features of senescent CD4^+^ T cells (59). The same group also found that terminally differentiated human CD8^+^ T cells (T_EMRA_) have decreased numbers of mitochondria and fail to efficiently upregulate glycolysis or OXPHOS following TCR activation, although showing high production of reactive oxygen species (48). These T_EMRA_ cells also showed elevated levels of p38 MAPK, and inhibition of p38 MAPK signalling elevated mitochondrial biogenesis and fitness. In addition, p38 MAPK blockade also induced an increase in autophagy through enhanced interactions between p38 interacting protein (p38IP) and autophagy protein 9 (ATG9) to compensate for the heightened energy demand (48). Of note, the polyamine spermidine is capable of inducing autophagy and undergoes an age-dependent decline (60). Recently, it was shown that spermidine modulates T cell differentiation towards a regulatory phenotype and dietary supplementation with spermidine reduced pathology in a mouse model of T cell transfer-induced colitis (61). Moreover, in a study in humans, spermidine supplementation was able to recover the autophagic flux and cellular functionality in T cells from old donors (62). Interestingly, spermidine was reported to reverse cellular senescence of B cells (63), an effect that has not yet been addressed in T cells.

## Discussion and Future Perspectives

Despite recent evidence suggesting an involvement of sTC in neurological diseases, many questions are still open. First, most studies analysing parameters such as telomere length and distribution of specific T cell subset are mainly observational, and do not investigate in detail the functional consequences of the observed changes. Second, identification of *bona fide* sTC may be tricky, due to overlapping expression of some senescence markers in effector memory *vs* terminally differentiated *vs* sTC (17, 18). Third, it is still not completely clear why patients with neurological disorders would accumulate sTC, compared to aged-matched individuals. Apart from the age-related reduction in the naïve T cells compartment, the main hypothesis is that circulating T cells undergo continuous antigen-specific or cytokine-induced re-activation, due to low grade systemic inflammation and/or to the presence of persistent CNS-derived antigens. This process would eventually reduce the extent of T cell receptor diversity, and increase the amount of terminally differentiated and potentially exhausted and senescent lymphocytes. Nonetheless, how immunosenescence develops and contributes to neuroinflammation and neurodegeneration remains unclear. Strikingly, a study showed that paediatric MS patients displayed signs of premature T cell senescence, with reduced number of circulating naïve T cells recently emigrated from the thymus, and increased percentage of memory-like cells, resembling the profile of adult individuals (24). This work may support the presence of an early antigen-specific (autoimmune) response inducing T cell maturation/activation, but might also suggest that appearance of a senescent/aged phenotype in T cells could predispose to MS development. Another aspect to consider is the recently hypothesised effect of disease-modifying therapies (DMT) on immunosenescence. Some anti-inflammatory DMT used to treat neuroinflammatory conditions, for example MS, may indeed not only limit the activation capacity of the immune system, but also induce signs of premature senescence of the immune system, such as reduced T cell output from the thymus (64, 65). These effects may differ between different DMT, potentially due to their intrinsic mechanisms of action (65). Thus, these DMT may be partially responsible for the appearance of severe side effects in aged individuals, such as viral reactivation and CNS inflammation and demyelination in MS patients (64, 65), again highlighting the importance of finely balancing aging of the immune system for optimal organismal homeostasis.

An increasing amount of studies showed metabolic alterations in circulating leukocytes in diseases of the CNS, with such metabolic modulation potentially playing a role in the pathogenic activity of different immune cell populations (66). However, direct comparisons of immunosenescence and immunometabolism in neurological diseases is lacking, even though the two features may be strongly interwoven. As an example, a recent work showed that T cells with dysfunctional mitochondria due to mitochondrial transcription factor A (TFAM) deficiency act as accelerators of senescence, and furthermore incite multiple aging-related features including neurological inflammation (67). This supports the notion that alterations of metabolic pathways in immune cells may directly cause inflammation-induced neurodegeneration. Importantly, there is a multitude of potential approaches to counteract age-associated immune cell malfunctions by metabolic intervention, including supplementation of age-limited nutrients such as formate and glycine, and reinforcement of autophagy with spermidine (47, 62). Of note, a recent study reported that mTOR inhibitor therapy in elderly humans decreased the incidence of infections, improved influenza vaccination responses and up-regulated antiviral immunity (68), thus highlighting the potential clinical relevance of such metabolic approaches in age-related immune dysfunctions. Given the therapeutic potential of immunometabolic intervention in neurological diseases (66), this strategy may indeed represent a brand-new approach to limit T cell senescence and dampen T cell-induced inflammation in CNS disorders and aging.

## Author Contributions

Both authors equally contributed to literature search and manuscript writing, and approved the final version of the manuscript.

## Conflict of Interest

The authors declare that the research was conducted in the absence of any commercial or financial relationships that could be construed as a potential conflict of interest.
